# Six-Minute Walk Test in Evaluation of Children with Pulmonary Arterial Hypertension 

**DOI:** 10.1007/s00246-017-1575-z

**Published:** 2017-02-27

**Authors:** Malgorzata Zuk, Anna Migdal, Dorota Jagiellowicz-Kowalska, Katarzyna Mazurkiewicz, Anna Sadel-Wieczorek, Grazyna Brzezinska-Rajszys

**Affiliations:** Department of Cardiology, The Children’s Memorial Health Institute, Al. Dzieci Polskich 20, 04-730 Warsaw, Poland

**Keywords:** Pulmonary arterial hypertension, Children, Six-minute walk test, Congenital heart disease

## Abstract

Six-minute walk test (6MWT) is a submaximal exercise test applied for evaluation of adults with pulmonary arterial hypertension (PAH). It was widely used as an endpoint in the clinical trials. The aim of the study was to assess the usefulness of 6MWT in management of children with PAH and to establish correlations with other clinical features. 164 6MWT were performed in 15 children between 5 and 18 years with PAH confirmed by right heart catheterization (102 in patients with shunt, 62 without shunt). Distance in 6MWT (6MWD)—% of predicted for age and gender, desaturation at the maximum effort, peak heart rate (HR)—% of maximal HR, were compared to the level of NTproBNP, WHO-FC, echocardiography parameters, and events of PAH treatment intensification. 6MWD had low negative correlation with peak HR (*τ* −0.1 *p* = 0,03), negative correlation with NTproBNP (*τ* −0.17 *p* = 0.002), and no dependence on echocardiography parameters. The presence of shunt was associated with lower 6MWD, lower blood saturation at rest, and higher desaturation after effort. Patients in III/IV WHO-FC achieved higher rest HR and maximal HR in comparison to patients in I/II WHO-FC (63.1 vs. 55.2% *p* < 0.01) and lower 6MWD (64.3 vs. 77.5% *p* < 0.01). In 14 out of 20 6MWT performed after treatment intensification, increase of distance was observed. The results of 6MWT were consistent with clinical status (WHO-FC, NTproBNP) but not with echocardiography parameters. 6MWT may be the source of additional information in management of children with PAH.

## Introduction

6-minute walk test (6MWT) is a submaximal exercise test applied for evaluation of adults with pulmonary arterial hypertension (PAH) [1]. It was widely used as an endpoint in the clinical studies [2]. According to European Society of Cardiology guidelines (2015 [1] and previous), distance reached in 6MWT (6MWD) is one of the factors in risk assessment in pulmonary arterial hypertension. In children, 6MWD is not used as a prognostic parameter [3] due to the difficult cooperation, dependence on age, height, and gender. However, it remains the only exercise test easy to perform and accepted by children with exercise limitations [4].

The aim of this study was to assess the usefulness of 6MWT for evaluation of children with pulmonary arterial hypertension and to establish correlations with other clinical features.

## Materials and Methods

**Fig. 1:**
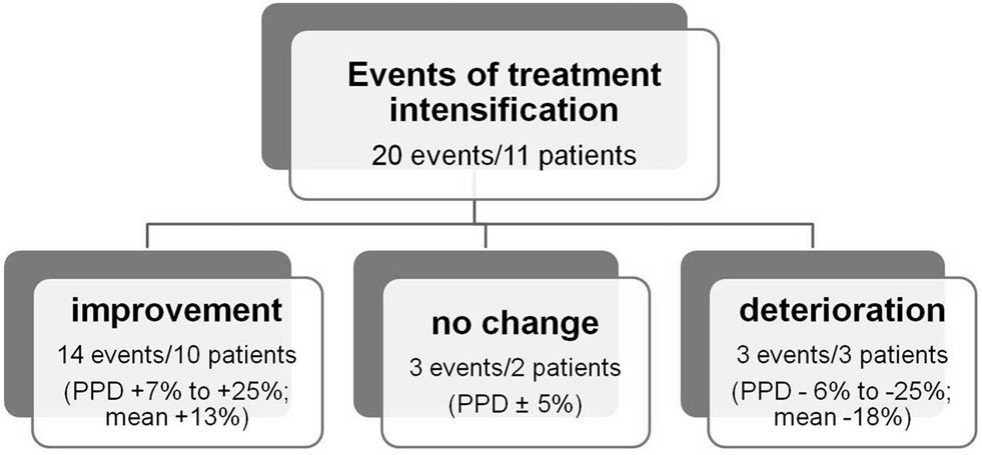
Change of 6MWD after treatment intensification (PPD—% of predicted distance for age and gender)

Analysis includes 164 6 MW tests performed every 3 months in 15 children with PAH confirmed in right heart catheterization. The number of tests per 1 patient ranged from 1 to 23 (mean 11 ± 7, median 9). The age during the test was 5–18 years (mean 13.4 ± 4.0; median 14.8), and there were no patients with Down syndrome and physical or mental disability. Distribution of tests performed in patients in I/II WHO functional class (WHO-FC) and III/IV WHO-FC was 129 to 35. 102 tests were done in patients with shunt—multiple ventricular septal defects, atrial septal defect *ostium secundum*, partial anomalous pulmonary venous drainage, tricuspid atresia, atrio-ventricular septal defect, idiopathic PAH after Potts shunt, and 62 in children without shunt—idiopathic PAH, postoperative congenital heart defect (Table 1). All patients had no bradycardia in 24-h Holter electrocardiography monitoring performed before 6MWT. The 6MWT were performed according to American Thoracic Society standard [5]. Distance, blood oxygen saturation before test (rest SAT) and desaturation at the maximum effort (rest SAT–SAT 6′), initial and maximal heart rate (rest HR, peak HR) were analyzed. Distance was shown as percentage of predicted for age and gender. Normal value of distance was calculated using gender-specific, age-adjusted reference equations [6] (Table 2). Heart rate was analyzed as percentage of median for age [7] (rest HR) and percentage of maximal heart rate for age (220-age)—peak HR. Submaximal exercise was defined as reaching no more than 85% of maximal heart rate [8]. Additionally ratio peak heart rate (% of max HR) to distance (% of predicted) was calculated. Obtained data were compared with the WHO-FC, level of N-terminal pro-brain natriuretic peptide (NTproBNP), echocardiographic parameters: left ventricular diastolic dimension—percentage of mean value for body surface area (LVDd%N), systolic gradient right ventricle-right atrium (∆RV/RA) (Table 3), and events of PAH treatment intensification (first administration, uptitration, or addition of new PAH-specific drug; Potts shunt) (Fig. 1). In order to determine the relationship between distance and clinical parameters, 6 MW tests were divided for three groups: with good, medium, and poor results according to percent of predicted distance (PPD >80%, 60–80%, and <60%) (Table 4).

**Table 1:** Clinical characteristics of the study population

ID	Gender	Diagnosis	Shunt	Number of 6MWT	First 6MWT				Last 6MWT				Δdistance	∆ PPD	Treatment intensification		
					Age	WHO-FC	NTproBNP (pg/ml)	Treatment	Age	WHO-FC	NTproBNP (pg/ml)	Treatment			Improvement	No change	Deterioration–intervention
1	F	IPAH		9	13.2	II	797	bos	15	IV	2428	bos. sild	−270	−41			1 − LTx
2	F	APAH CHD	AVSD	15	14.7	III	220		18	II	365	bos	121	23	2		
3	F	IPAH		16	14.8	III	2988		17.9	II	60	bos. tad	225	39	2		
4	F	HPAH		12	16.3	III	1144		18	III	1368	bos. sild. ilo	15	4	2	2	
5	M	APAH CHD	mVSD	23	8.7	II	170	bos. sild	15.8	II	29	bos. sild	120	9			
6	M	APAH CHD postop		8	7.2	II	235		9	II	118	bos	131	17	1		
7	F	IPAH	Potts shunt*	6	5.1	IV	17,272	bos. sild. epo	5.8	III	6557	bos. sild. Potts	10	0	1		1 − Potts shunt
8	F	APAH CHD	ASD II	23	6.4	II		sild	13.5	II	306	bos. sild	60	−3	1		
9	F	APAH CHD	AT	7	16.7	II	122		18	II	168	bos	42	8	1		
10	F	IPAH		15	13.1	II		sild	18	II	60	bos	95	20	1		
11	F	IPAH		1	16.1	IV	5000	Death after RHC									
12	F	APAH CHD	ASD II	15	14.6	II	316		18	III	194	bos. sild	−90	−11	1		
13	M	APAH CHD	ASD. PAPVD	7	6.4	III	1908		7.7	III	4718	bos. sild	185	31	2	1	1 − dose escalation
14	F	IPAH	Potts shunt	4	5.4	I	78	bos. sild. Potts	6.1	I	109	bos. sild. Potts	−31	−8			
15	F	APAH CHD	mVSD	3	5	II	192	bos	5.4	II	206	bos	30	5			

*6MWT* six-minute walk test, *PPD* percent of predicted distance, *IPAH* idiopathic pulmonary arterial hypertension, *HPAH* heritable pulmonary arterial hypertension, *APAH* pulmonary arterial hypertension associated with, *CHD* congenital heart defect, *CHD postop* congenital heart defect after shunt closure, *AVSD* atrio-ventricular septal defect, *mVSD* multiple ventricular septal defects, *ASD II* atrial septal defect *ostium secundum, AT* tricuspid atresia, *PAPVD* partial anomalous pulmonary venous drainage, *RHC* right heart catheterization, *bos* bosentan, *sild* sildenafil, *tad* tadalafil, *ilo* iloprost, *epo* epoprostenol, *LTx* lung transplantation

*1st test before Potts shunt

**Table 2:** Equations to predict the 6MWD in children and adolescents—age-adjusted model [6]

Boys	
<13 yo	24.18 × *y* + 385.18
>13 yo	13.08 × *y* + 476.69
Girls	
<12 yo	20.83 × *y* + 413.94
>12 yo	−8.66 × *y* + 757.42

**Table 3:** Results of all analyzed 6MWT (data presented as mean ± SD and median)

Parameters	Units	All 6MWT	Shunt	No shunt	*p*	WHO-FC I/II	WHO-FC III/IV	*p*
***N***		**164**	**102**	**62**		**124**	**40**	
6MWD	% of predicted	74.3 ± 12.6 76.5	**72.8** ± **12.4** **75.2**	**76.9** ± **12.5** **79.3**	**0.02**	**77.5** ± **8.9** **78.0**	**64.3** ± **16.4** **70.6**	<**0.01**
Rest SAT	%HbO2	92.6 ± 6.3 96	**89.6** ± **6.4** **91**	**97.5** ± **1.1** **98**	<**0.01**	93.1 ± 5.7 6.0	90.9 ± 7.8 95.0	NS
Desaturation	%HbO2	11.8 ± 11.2 10.0	**17.5** ± **10.4** **17.5**	**2.6** ± **4.3** **1**	<**0.01**	11.8 ± 11.4 10.0	11.7 ± 10.6 9.0	NS
Rest HR	% of median for age	101 ± 21 101	99.3 ± 2.119 9.3	102.9 ± 18.6 103.3	NS	**96.6** ± **20.9** **96.5**	**113.1** ± **18.5** **110.3**	<**0.01**
Peak HR	% of max HR for age	57.1 ± 11.9 56.9	57.3 ± 11.1 58.2	56.9 ± 13.3 54.9	NS	**55.1** ± **11.2** **54.3**	**63.1** ± **12.35** **9.4**	<**0.01**
Peak HR/6MWD		0.79 ± 0.33 0.75	0.81 ± 0.35 0.77	0.77 ± 0.29 0.7	NS	**0.71** ± **0.2** **0.7**	**1.12** ± **0.56** **1.05**	<**0.01**
NTproBNP	pg/ml	875 ± 1904 255	804 ± 1612 271	995 ± 2336 176	NS	**244** ± **244** **165**	**2766** ± **3120** **1832**	<**0.01**
ΔRV-RA	mmHg	95 ± 25 100	**101.7** ± **21.8** **103**	**83.9** ± **25** **82**	<**0.01**	94.2 ± 27.5 101.0	95.8 ± 17 90.5	NS
LVDd %N	% of mean normal value	90.8 ± 11.5 92	92.8 ± 10.5 94	88.7 ± 12.3 91.5	NS	91.1 ± 10.9 93.0	90.2 ± 12.1 91.0	NS
Hb	g/l	146 ± 18 149	**152** ± **15** **152**	**136** ± **17** **132**	<**0.01**	145 ± 19 149	149 ± 14 152	NS

Bold values indicate statistically significant (*p* < 0.05)

**Table 4:** 6MWTs divided into groups: good, medium and poor result (data presented as mean ± SD*)*

Group	All *N* = 164 Mean PPD 74.3 ± 12.6%	Good PPD >80% *N* = 51 Mean PPD 85.6 ± 5.2%	Median PPD 60–80% *N* = 93 Mean PPD 73.8 ± 4.8%	Poor PPD <60% *N* = 20 Mean PPD 48.1 ± 10.1%	*p*
Clinical data					
Shunt	102 (62%)	23 (45%)	64 (69%)	15 (75%)	<0.01
Age (years)	13.4 ± 4.0	14.7 ± 3.4	13.5 ± 3.6	9.4 ± 4.6	<0.01
WHO-FC	2.2	1.9	2.2	2.9	<0.01
Rest HR	80.0 ± 17.7	77.2 ± 14.2	75.9 ± 13.6	106.3 ± 20.7	<0.01
Rest SAT (%HbO2)	92.6 ± 6.3	93.9 ± 5.7	92.8 ± 5.9	88.9 ± 8.2	<0.01
NTproBNP (pg/ml)	899.9 ± 1946.9	431.1 ± 762.2	666.9 ± 1952.8	3108.5 ± 2495.4	<0.01
Echo					
LVDd%N	90.8 ± 11.5	89.5 ± 9.9	91.4 ± 12.3	90.8 ± 11.6	NS
ΔRV-RA	94.7 ± 24.7	88.7 ± 26.9	96.6 ± 25.4	96.2 ± 19.3	NS
6MWT					
Peak HR	117.9 ± 24.4	114.0 ± 23.3	117.2 ± 25.0	131.4 ± 20.5	0.02
HR% max	57.1 ± 11.9%	55.6 ± 11.4%	56.8 ± 12.4%	62.5 ± 10.0%	NS
ΔHR	38.6 ± 25.1	36.8 ± 24.0	42.3 ± 24.1	25.5 ± 28.5	0.02
Peak SAT (%HbO2)	80.7 ± 14.6	85.5 ± 13.7	78.4 ± 14.5	78.7 ± 14.6	0.01
Desaturation (%HbO2)	11.8 ± 11	7.8 ± 9.7	14.4 ± 11.7	10.2 ± 9.5	<0.01

Abbreviations description in the text

 Procedures analyzed in this study are the part of standard care of patients with pulmonary hypertension. All are required by the National Health Fund to reimbursement of pharmacotherapy. Informed consent to participate in the therapeutic program of the National Health Fund was obtained in all patients.

### Statistical Analysis

Continuous data were presented as values with an average, a standard deviation, and a median. Normal distribution was determined using Shapiro–Wilk test. To compare of distribution in groups, parametric (T-student) and non-parametric (Whitney–Mann) tests were used. The limit for statistical significance was set at *p* < 0.05. Obtained data were shown on the boxplot. Differences among three groups of tests (good, medium, poor result) were analyzed using analysis of variance (ANOVA). To measure the association between two measured quantities, Kendall’s rank correlation coefficient (tau) was used.

## Results

 In all tests, reached distance adjusted to age and gender ranged from 22 to 109% of predicted value (mean 74.3 ± 12.6%; median 76.5%). 11 patients in 51 tests reached distance more than 80% predicted (good result group). In this group, a lower WHO-FC (92% WHO-FC I/II) and a lower level of NTproBNP (431.1 ± 762.2) were found. 55% of tests were performed by patients with PAH without shunt therefore with higher saturation, and lower mean desaturation. 7 patients in 20 tests did not achieve 60% PPD (poor result group). Mean age during tests was lower than in other groups. 75% tests in this group were performed by patients with shunt therefore the rest saturation was lower. Patients were in higher WHO-FC (80% WHO-FC III/IV), with higher level of NTproBNP (3108.5 ± 2495.4), rest HR, and peak HR despite the increase heart rate (ΔHR) was lower. There were no differences in echocardiography parameters irrespectively of 6MWT results (good, medium, and poor).

**Fig. 2:**
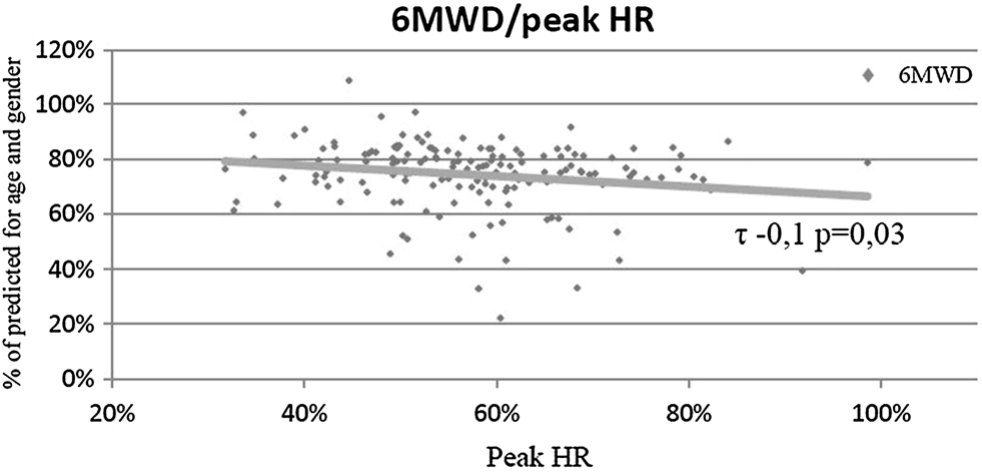
Correlation between 6MWD and peak heart rate (peak HR)

**Fig. 3:**
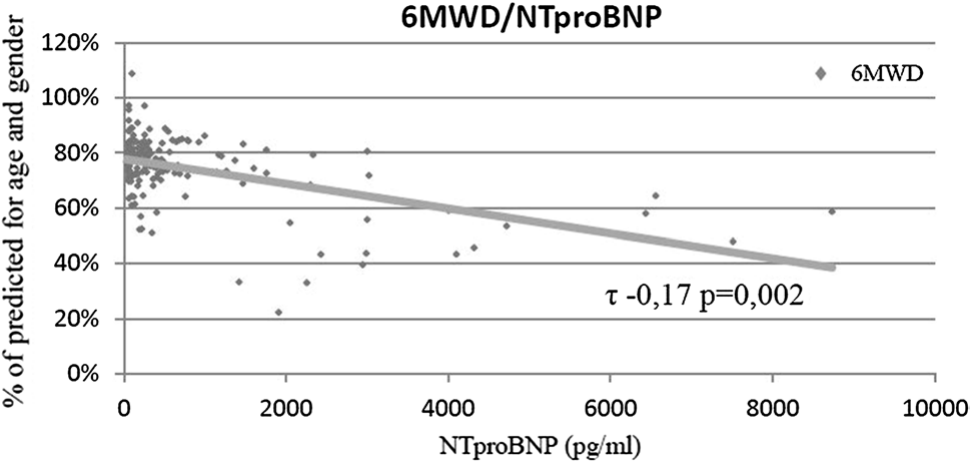
Correlation between 6MWD and NTproBNP level

In whole material, the walk distance had low negative correlation with peak heart rate (*τ* −0.1 *p* = 0.03) (Fig. 2) and negative correlation with NTproBNP (*τ* −0.17 *p* = 0.002) (Fig. 3). No dependence on echocardiography parameters was found. All results of 6MWT are shown in (Table 4).

20 events of treatment intensification were detected in 11 patients (Fig. 1). In 17 cases, it was associated with at least 5% change of distance in the next 6MWT (after 1–3 months). Increase in percentage of predicted value (7–25%, mean 13 ± 6, median 10.5%) was observed in 14 events in 10 patients. In 3 patients, the percentage of predicted value decreased by 6–25%. In those cases further intensification of treatment was taken: dose escalation with good results in 1 patient, Potts shunt in 1 and lung transplantation in 1 (Table 1).

### Shunt Versus No Shunt (Table 3)

**Fig. 4:**
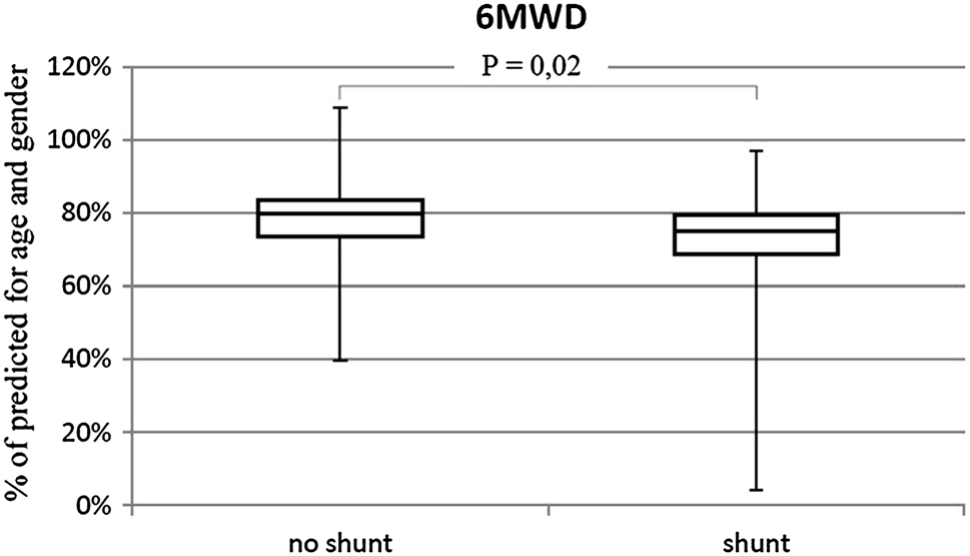
6MWD in patients with shunt and no shunt

**Fig. 5:**
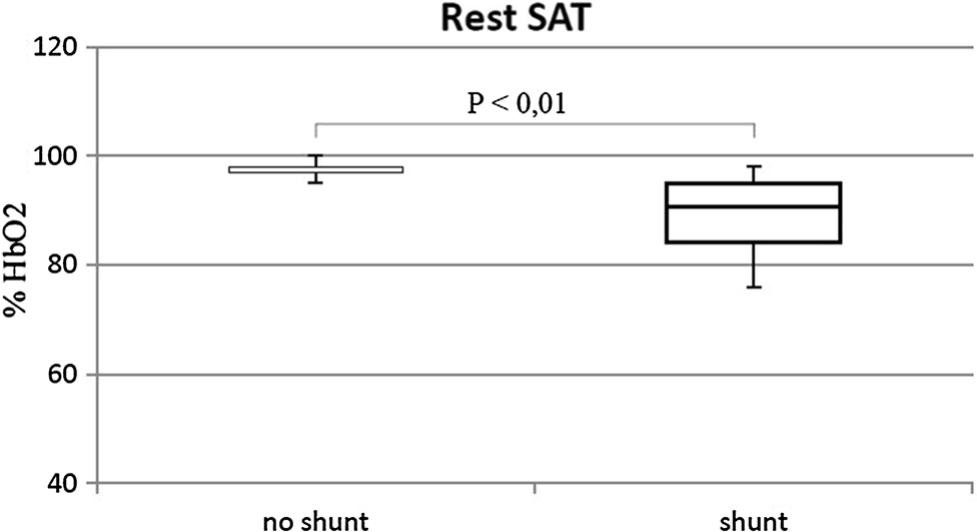
Blood saturation at rest (rest SAT) in patients with shunt and with no shunt

**Fig. 6:**
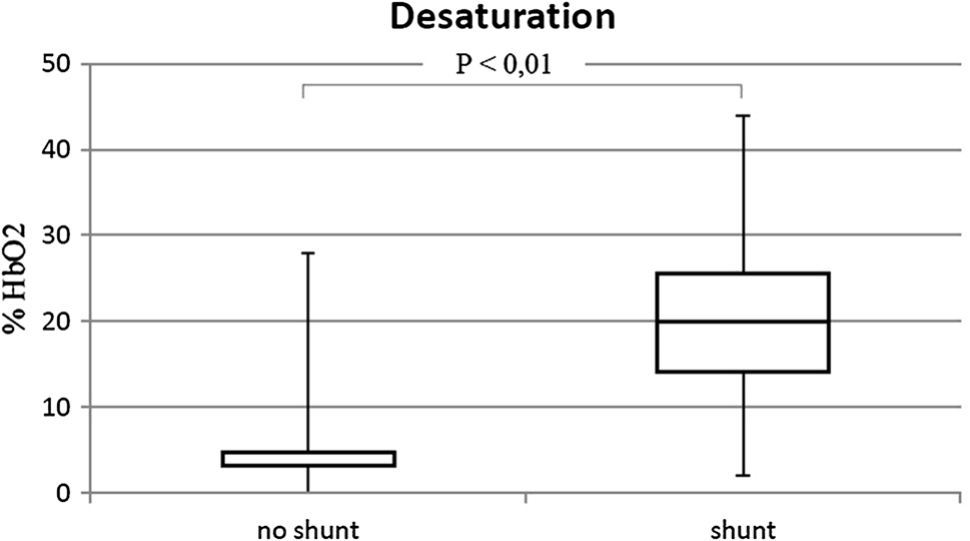
Desaturation after effort in patients with shunt and with no shunt

The percentage of patients in WHO-FC III/IV was similar in both groups (shunt 27.2%; no shunt 22.5%). Walk distance in patients with shunt was lower than without shunt (73 vs. 77% *p* = 0.02) (Fig. 4). No differences in heart rate (initial and peak) were observed. The presence of shunt was associated with lower blood saturation at rest (90 vs. 98% *p* < 0.01) (Fig. 5) and higher desaturation after effort (17.5%HbO2 vs. 3%HbO2 *p* < 0.01) (Fig. 6).

### WHO III/IV Versus I/II (Table 3)

**Fig. 7:**
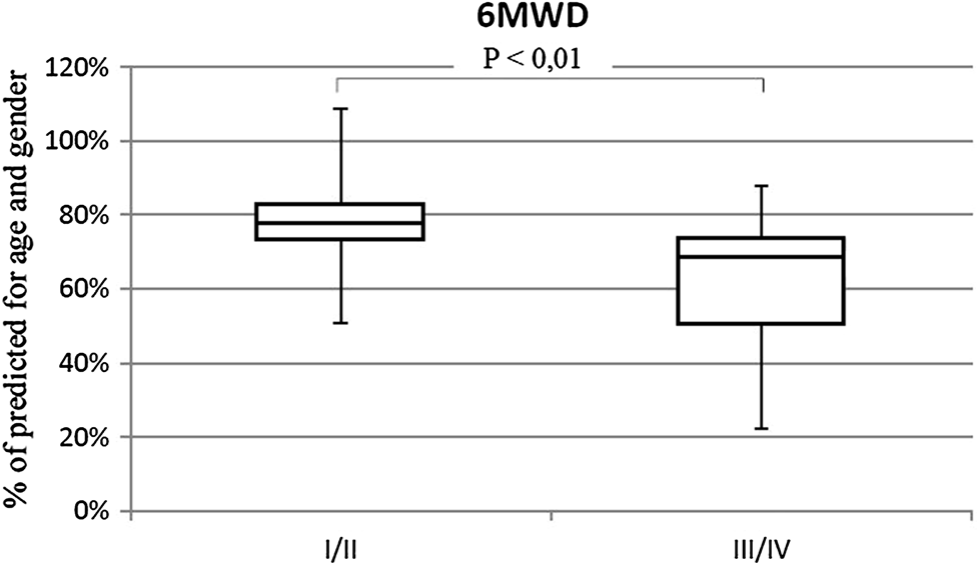
6MWD in patients in I/II WHO-FC in comparison to patients in III/IV WHO-FC

**Fig. 8:**
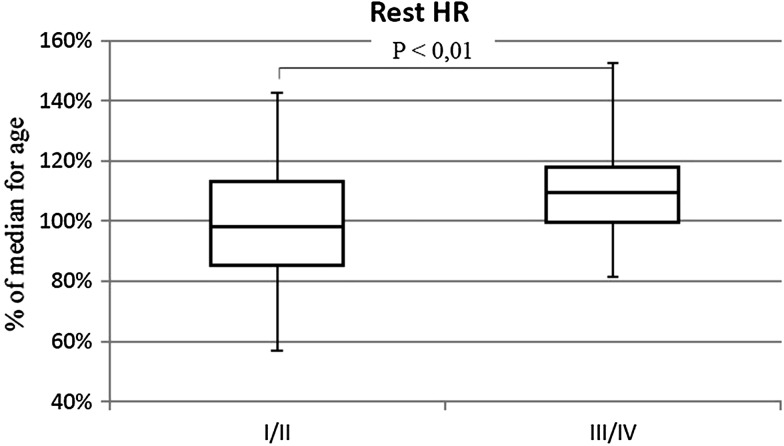
Rest heart rate (rest HR) in patients in I/II WHO-FC in comparison to patients in III/IV WHO-FC

**Fig. 9:**
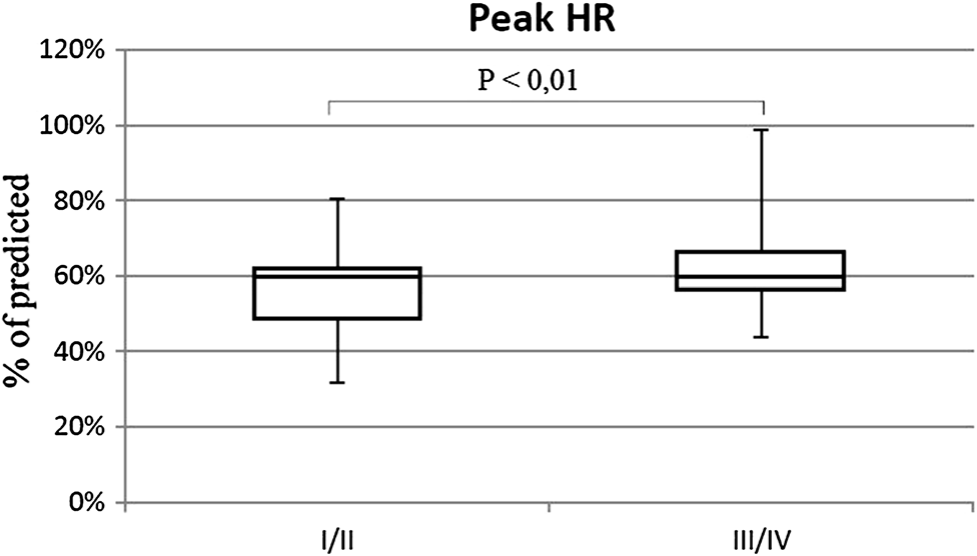
Peak heart rate (peak HR) in patients in I/II WHO-FC in comparison to patients in III/IV WHO-FC

**Fig. 10:**
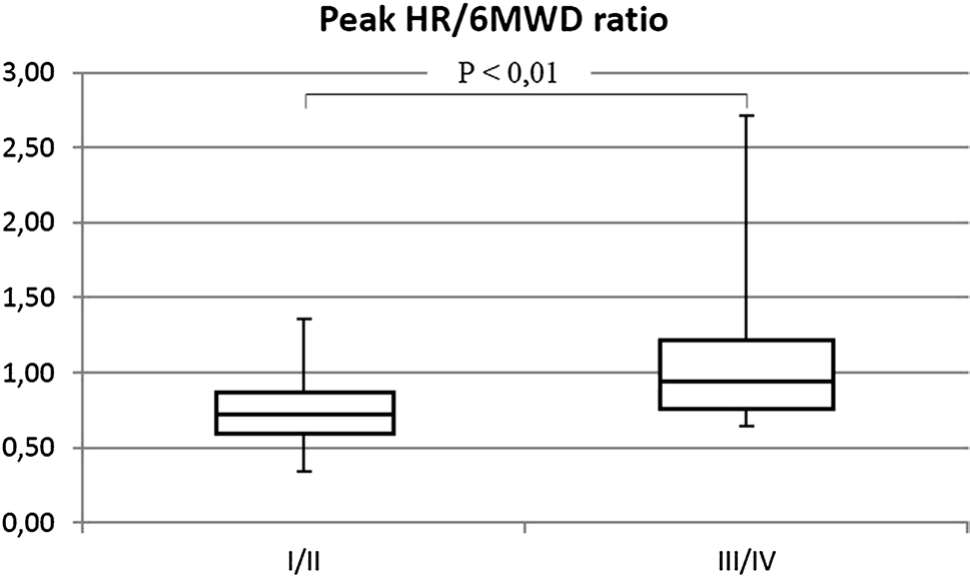
Peak heart rate (peak HR) to 6MWD ratio in patients in I/II WHO-FC in comparison to patients in III/IV WHO-FC

The percentage of patients with shunt was similar in both groups (III/IV 57.5%; I/II 63.7%). Patients in III/IV WHO-FC achieved significantly lower walk distance than children with I/II WHO-FC (64.3 vs. 77.5% *p* < 0.01) (Fig. 7). They had higher rest HR (113 vs. 97% of normal value *p* < 0.01) (Fig. 8), similar increase of HR during effort (38 vs. 39 beats NS), and higher peak HR (63.1 vs. 55.2% of maximal heart rate for age *p* < 0.01) (Fig. 9). Only in six tests criteria of submaximal exercise was exceeded or was only slightly below limit (81–84%). Five of them were performed by patients in III WHO-FC with the average distance 69 vs. 74% in the rest of the tests (too small group for statistical analysis). Peak heart rate-to-distance ratio in patients with III/IV WHO-FC was significantly higher than in patients in I/II WHO-FC (1.1 vs. 0.7 *p* < 0.01) (Fig. 10).

## Discussion

This study demonstrated that the results of 6MWT in children with pulmonary hypertension are associated with clinical status, especially with WHO-FC and NTproBNP which are unquestioned prognostic factors in this disease [3]. No dependence on echocardiography parameters was detected, but only basic measurements were included. Similarly Placido et al. [9] reported no correlation between conventional parameters of the RV function and 6 MWD in adults with pulmonary arterial hypertension. It is possible that more detail examination of systolic and diastolic function in echo using advanced methods will be more useful to detect correlation between echo and 6MWD and this needs further investigation, especially in children.

The usefulness of the 6MWT was confirmed in many studies in adults [10–12], so in ESC guidelines 2015 [1] and previous, 6MWD is recommended as one of prognosis determinants. There are many doubts regarding the usefulness of 6MWT in children because the results depend on age, gender, and developmental parameters. Additionally, reproducibility of this test in children depends on proper cooperation, which is not always possible. In our study, we use percentage of normal value adjusted for age and gender to eliminate developmental differences. Martins et al. [13] have verified the reproducibility of the 6-min walk test in healthy children at the age of 6–14 years. No effect of learning on the distance in second test was observed. Similar results were obtained by Morinder et al [14], who examined a group of 49 obese and 97 healthy children. Taking into account the above evidence, analysis of the individual tests (not individual patients) seems reasonable and should not be fraught with bias resulting from age and the effect of learning.

Many authors evaluated the results of 6MWT in children with chronic diseases (for example, pulmonary diseases [15], obesities [14], connective tissue diseases, and hematological and neurological abnormalities [16]). In most studies, 6MWT was recognized as a useful in the evaluation of chronically ill children. However, standards of performance of 6MWT are different, so comparison between studies is difficult and conclusion should be interpreted with caution [17].

There are limited data concerning the usefulness of 6MWT in children with PAH.

Based on recent studies, where baseline 6MWD was not a predictor of survival in children with pulmonary hypertension, Ivy et al. [18] concluded that 6MWT should not be consider in risk assessment in PAH children in contrast to adults. However, the results of other studies suggest that 6MWT can be useful in the management of pediatric PAH. Lammers et al. [19] published data of 47 children with PAH at the mean age of 11.4 years, where 6 MW distance in single measurement was one of the predictors of death or need for transplantation. The same author [20] evaluated results of 6MWT in PAH children. In this study, 6MWD was significantly shorter than predicted (47.7 ± 16.7%). 6MWT was a submaximal test in most of patients. Heart rate limit for maximal effort was reached only by children with a 6MWT distance of less than 300 m. That is compatible with our observation—distance in all tests was less than predicted, and criteria of submaximal effort exceeded patients with WHO-FC III/IV with shorter 6MWD.

We observed shorter 6MWD in PAH patients with shunt compare to patients with IPAH or PAH after shunt closure. The same results, but without statistical significance, were published by Lammers [20] and Douwes [21]. Additionally, Lammers demonstrated lower exercise capacity measured in CPET in patients with Eisenmenger syndrome compare to patient after shunt closure. As explained by Dimopoulos [22], it may be abnormal ventilatory response to exercise due to cyanosis. In the presented material, patients with Eisenmenger syndrome characterized by ventilation to perfusion mismatch. They have lower peak oxygen uptake (VO2max) and significantly higher ventilation per unit of CO2 production (VE/VCO2) slope, and lower expiratory exchange ratio at peak exercise.

Study published by Douwes [21] contains two issues—evaluation of 6MWT as a prognostic factor and as a determinant of disease severity. 6MWD was negatively correlated with NTproBNP and WHO-FC (similarly to our results and Van Albada study [23]). Patients with shunt had higher desaturation on the peak of effort. No correlation between heart rate and WHO-FC was observed. On the contrary, our study showed higher heart rate during 6MWT, baseline and maximal, in patients in III/IV WHO-FC. The results of both studies (Douwes’ and ours) conducted using different methods indicate that 6MWT may be useful in monitoring of disease severity.

## Study Limitation

We analyzed the tests independently which were repeated several times by the same patients, however, in different clinical conditions. The study group does not have limitations associated with the physical development or intellectual disability or pathology of other systems. The bias related to age and the effect of learning seems to be irrelevant of the reasons mentioned above. However, the individual differences in exercise capacity of patients may affect the results. On the other hand, the studies assessing 1 test in each patient do not take into account the changes of clinical status and treatment. Thus, the predictive value of identical result of the test in child before treatment vs child receiving the combination therapy is different. Therefore it would be rational to apply advanced statistical modeling methods in a larger group of patients who have performed many tests to achieve more satisfactory results.

## Conclusions

The results of the 6MWT in children with pulmonary hypertension reflect clinical status (WHO-FC, NTproBNP), but not echocardiography parameters. 6MWT may be the source of additional information in assessment and management of children with PAH. 6MWT repeated in routine control may be helpful in treatment decisions.

## Abbreviations

6MWT: 6-minute walk test
6MWD: 6-minute walk distance
HR: Heart rate
LVDd%N: Left ventricular diastolic dimension—percentage of mean value for body surface area
NTproBNP: N-terminal pro-brain natriuretic peptide
PAH: Pulmonary arterial hypertension
∆RV/RA: Systolic gradient right ventricle-right atrium
SAT: Blood oxygen saturation
WHO-FC: WHO functional class
